# Letter to the Editor: Comments on statistical methods and interpretation in surgical strategy of retroperitoneal liposarcoma involving the kidney capsule

**DOI:** 10.1097/MS9.0000000000002810

**Published:** 2024-12-20

**Authors:** Guang Xiong

**Affiliations:** Department of Geriatric Endocrinology and Metabolism, The First Affiliated Hospital of Guangxi Medical University, Guangxi, China

*Dear Editor*,

I read with great interest the article “Clinical features and surgical strategy of retroperitoneal liposarcoma involving the kidney capsule: a retrospective comparative cohort study” by Li *et al*^[1]^. I would like to express my appreciation for the authors’ valuable research contributions. However, I have several queries concerning the statistical methods and interpretation of the study’s results. I believe a more detailed discussion on the study design and outcomes is necessary, and I kindly request clarification from the authors.

In Table 3, I noticed a negative hazard ratio (HR). According to the statistical definition, HR represents the risk ratio between an exposed group and an unexposed group, indicating the relative risk of an event between these groups^[2]^. Therefore, HR values should always be positive^[2]^. The presence of a negative HR may suggest errors in either calculation or presentation. Clarification on the method used to calculate the HR and an explanation for the negative value are necessary. Additionally, in Table 3, the authors analyzed the impact of nephrectomy (a categorical variable) on postoperative eGFR (a continuous variable) and performed a stratified analysis. Conceptually, such data should be examined using a linear regression model, with the effect size expressed as a regression coefficient^[3]^. The statistical model applied was not specified in the article. Providing details on whether a linear regression model was used, along with an explanation of the statistical approach chosen and its underlying assumptions, would enhance the clarity of the analysis.

Regarding Figure 3, the Kaplan-Meier survival curves intersect multiple times, which indicates that the relative risk between groups changes over time, thereby violating the proportional hazards assumption of the Log-Rank test^[4]^. The validity of the Log-Rank test relies on this assumption, meaning that the hazard ratio between groups remains constant over time. If this assumption does not hold, the Log-Rank test may lack sufficient statistical power to detect differences between groups^[4]^. Consequently, the results obtained may be misleading and could lead to incorrect conclusions^[4]^. Additionally, we observed in Figure 3B that the nephrectomy group initially had a higher survival rate than the non-nephrectomy group, with the KM curves crossing around 60 months. This suggests that the nephrectomy group may have had better survival before 60 months. Continuing to use the Log-Rank test under such circumstances could lead to misleading or inaccurate results. To enhance the reliability of the findings, I recommend the authors apply a weighted Cox regression model, which accommodates time-varying effects^[5]^. This approach would offer an average HR estimate that is not contingent upon the proportional hazard assumption, providing a more accurate depiction of the risk dynamics between groups over time^[5]^. The Royston–Parmar flexible parametric model may also be useful to explore the trend of risk ratios over time, offering a more intuitive description of how the risk evolves^[5]^.

Lastly, the authors concluded that nephrectomy is linked to a higher risk of postoperative acute kidney injury (AKI), and they advocate for a kidney-sparing approach to achieve optimal clinical outcomes. However, the survival analysis does not indicate a significant difference in survival rates between the nephrectomy and non-nephrectomy groups, which challenges the assertion that renal preservation yields the best clinical outcomes. Additionally, although nephrectomy is associated with an elevated risk of postoperative AKI, focusing solely on AKI incidence may not sufficiently evaluate long-term renal function prognosis. I suggest that, to better assess the long-term renal impact, the authors should compare long-term eGFR to provide a more comprehensive analysis.

I greatly appreciate the authors’ valuable contributions to this field, and I hope they can kindly address these points in detail to further enhance the understanding and application of their conclusions.

## Data Availability

Not applicable.
